# The Potent Trypanocidal Effect of LQB303, a Novel Redox-Active Phenyl-Tert-Butyl-Nitrone Derivate That Causes Mitochondrial Collapse in *Trypanosoma cruzi*

**DOI:** 10.3389/fmicb.2021.617504

**Published:** 2021-04-15

**Authors:** Carolina Machado Macedo, Francis Monique de Souza Saraiva, Jéssica Isis Oliveira Paula, Suelen de Brito Nascimento, Débora de Souza dos Santos Costa, Paulo Roberto Ribeiro Costa, Ayres Guimarães Dias, Marcia Cristina Paes, Natália Pereira Nogueira

**Affiliations:** ^1^Laboratório de Interação de Tripanossomatídeos e Vetores, Departamento de Bioquímica, IBRAG – Universidade do Estado do Rio de Janeiro, Rio de Janeiro, Brazil; ^2^Laboratório de Hematologia, Departamento de Patologia, Faculdade de Medicina, Universidade Federal Fluminense, Rio de Janeiro, Brazil; ^3^Departamento de Química Orgânica, Universidade do Estado do Rio de Janeiro, Rio de Janeiro, Brazil; ^4^Laboratório de Química Bioorgânica, NPPN, Universidade Federal do Rio de Janeiro, Rio de Janeiro, Brazil; ^5^Instituto Nacional de Ciência e Tecnologia - Entomologia Molecular (INCT-EM), Rio de Janeiro, Brazil

**Keywords:** *Trypanosoma cruzi*, chemotherapy, bioenergetics, high-resolution respirometry, mitochondrion, tropical neglected disease

## Abstract

Chagas disease, which is caused by *Trypanosoma cruzi*, establishes lifelong infections in humans and other mammals that lead to severe cardiac and gastrointestinal complications despite the competent immune response of the hosts. Furthermore, it is a neglected disease that affects 8 million people worldwide. The scenario is even more frustrating since the main chemotherapy is based on benznidazole, a drug that presents severe side effects and low efficacy in the chronic phase of the disease. Thus, the search for new therapeutic alternatives is urgent. In the present study, we investigated the activity of a novel phenyl-tert-butyl-nitrone (PBN) derivate, LQB303, against *T. cruzi*. LQB303 presented trypanocidal effect against intracellular [IC_50_/48 h = 2.6 μM] and extracellular amastigotes [IC_50_/24 h = 3.3 μM] *in vitro*, leading to parasite lysis; however, it does not present any toxicity to host cells. Despite emerging evidence that mitochondrial metabolism is essential for amastigotes to grow inside mammalian cells, the mechanism of redox-active molecules that target *T. cruzi* mitochondrion is still poorly explored. Therefore, we investigated if LQB303 trypanocidal activity was related to the impairment of the mitochondrial function of amastigotes. The investigation showed there was a significant decrease compared to the baseline oxygen consumption rate (OCR) of LQB303-treated extracellular amastigotes of *T. cruzi*, as well as reduction of “proton leak” (the depletion of proton motive force by the inhibition of F1Fo ATP synthase) and “ETS” (maximal oxygen consumption after uncoupling) oxygen consumption rates. Interestingly, the residual respiration (“ROX”) enhanced about three times in LQB303-treated amastigotes. The spare respiratory capacity ratio (SRC: cell ability to meet new energy demands) and the ATP-linked OCR were also impaired by LQB303 treatment, correlating the trypanocidal activity of LQB303 with the impairment of mitochondrial redox metabolism of amastigotes. Flow cytometric analysis demonstrated a significant reduction of the ΔΨm of treated amastigotes. LQB303 had no significant influence on the OCR of treated mammalian cells, evidencing its specificity against *T. cruzi* mitochondrial metabolism. Our results suggest a promising trypanocidal activity of LQB303, associated with parasite bioenergetic inefficiency, with no influence on the host energy metabolism, a fact that may point to an attractive alternative therapy for Chagas disease.

## Introduction

Chagas disease (CD), which is caused by the protozoan *Trypanosoma cruzi*, is the most important neglected tropical disease in the world, and it is a major social and public health problem in Latin America, according to the World Health Organization (WHO; Rassi et al., 2010; Pérez-Molina and Molina, 2018). It is estimated that 7 million people worldwide are infected with *T. cruzi* and due to globalization and migratory movements, it is estimated that around 75 million people are at risk of contracting the disease (WHO, 2020). Today, less than 10% of people with the disease are diagnosed and only 1% of them receive adequate treatment (Ribeiro et al., 2009). CD is defined by two phases, acute and chronic. The acute phase is characterized by high parasitemia and asymptomatic. About 30% of those infected develop the chronic phase of the disease and are characterized by indeterminate and asymptomatic clinical forms, cardiac, and digestive forms (Coura and Borges-Pereira, 2011; Echeverria and Morillo, 2019).

The *Trypanosoma cruzi* has a complex life cycle, alternating between the insect vector triatomine and the mammalian host. During its life cycle, *T. cruzi* presents four main stages: epimastigote and metacyclic trypomastigotes in the insect vector and the clinically relevant forms of the parasite, amastigotes, and bloodstream trypomastigotes in vertebrate hosts, which alternate through differentiation processes described as epimastigogenesis, metacyclogenesis, amastigogenesis, and trypomastigogenesis, respectively (Contreras et al., 2002; Hernández-Osorio et al., 2010; Kessler et al., 2017).

Among these processes, amastigogenesis is the differentiation from trypomastigotes into amastigotes that occurs inside vertebrate host cells after invasion and can be mimicked *in vitro* by low pH. Extracellular amastigotes (EAs) are believed to be generated not only by extracellular differentiation of trypomastigotes, but also by premature lysis of infected host cells. EAs are infective and able to invade a variety of mammalian cells (Ferreira et al., 2012). Dormant *T. cruzi* amastigotes occur spontaneously in the host tissues, but this condition does not seem to be a response to a lack of nutrients or the presence of stressors such as the host immune system. In addition, they resist being killed by various compounds that are highly active against replicating parasites (Sánchez-Valdéz et al., 2018; Barrett et al., 2019).

Although it was first described 112 years ago by the Brazilian researcher Carlos Chagas, the only treatments currently available for Chagas disease involve two nitroheterocyclic drugs (benznidazole and nifurtimox), which are unsatisfactory. This is due to their high toxicity and severe side effects, in addition to having low efficacy in the chronic phase of the disease and the natural resistance observed in some strains of the parasite (Urbina, 2010; Rassi et al., 2012; Bahia et al., 2014). Furthermore, several challenges remain to be overcome besides the development of new drugs. First of all, there is the need for proper access to diagnostics and the tools to assess parasitological cure. Then, there are the variety of unanswered questions regarding both the disease itself and parasite-host interactions, and finally, there is the optimization of the available antitrypanosomal drugs, which require long-term treatment, and even then, are unable to eliminate all parasites in some patients. There is, therefore, a clear need for new safe, effective, and accessible therapies for CD (Chatelain, 2015).

Unlike mammalian cells, *T. cruzi* displays a single mitochondrion with several unique features, such as a peculiar arrangement of the kinetoplast (mitochondrial DNA) and the presence of enzymes that participate in both the energy metabolism and antioxidant network. During the *T. cruzi* life cycle, the shape and functional plasticity of the single mitochondrion undergoes profound alterations, reflecting adaptations to different environments faced by the parasites (Paes et al., 2011). The mitochondrial metabolism of *T. cruzi* involves a variety of enzymes and proteins, connected to many metabolic processes. While the bioenergetic role of the mitochondrion is linked to the electron transport system and enzymes for oxidative phosphorylation, there are several important biosynthetic activities that take place in this organelle (Monzote and Gille, 2010). Furthermore, the mitochondrial differences between mammals and trypanosomatids make this organelle an excellent candidate for drug intervention.

Chemically, nitrones are organic molecules with activated carbon nitrogen double bond [R^1^R^2^**C=N**^+^ (**O**^−^)R^3^] and are able to trap and scavenge reactive oxygen/nitrogen species such as superoxide radical, hydrogen peroxide, nitric oxide, or peroxynitrite. Consequently, nitrones were initially used because they have extremely potent activity in biological systems, mainly due to their free radical “spin trap” capacity. Subsequent studies have shown that alpha-phenyl-*N*-*tert*-butylnitrone(PBN) nitrone, as well as its analogs demonstrated low toxicity and potent pharmacological activity against Parkinson’s disease and Alzheimer’s (Floyd et al., 2008, 2013) and ischemic stroke (Contelles, 2020).

In experimental research, epimastigotes, the replicative stage present in the insect vector are often used for the activity of new compounds, due to the availability and fast proliferation of their axenic cultures. However, epimastigotes are not directly involved in the pathology of the disease and the data obtained cannot always be extrapolated to other stages of development. Therefore, the use of clinically relevant forms of *T. cruzi* for human infection, such as amastigotes is necessary for the evaluation of new compounds (Romanha et al., 2010; Miranda et al., 2015).

The literature shows that the combination of PBN and benznidazole (BZ) decreased the level of mitochondrial reactive oxygen species (ROS) production and chronic heart failure in chagasic mice. However, mice treated with PBN alone did not show decreased parasite persistence (Wen et al., 2010). Modifications to the PBN molecule increases its trypanocidal effect, as seen with the PBN derivative, LQB123, which demonstrates trypanocidal effects on the proliferative and infective forms of *T. cruzi*, as well as low cytotoxicity to mammalian cells (Cupello et al., 2017).

In this present work, we evaluated the activity of the PBN derivative, LQB303, and its direct impact on the proliferative form of the mammalian host, amastigotes, as well as its mitochondrial physiology and bioenergetics.

## Materials and Methods

### Compound

LQB303 was synthesized according to Costa (2020). Stock solutions of LQB303 were prepared in dimethyl sulfoxide (DMSO, MERCK, United States), and the final concentration of the solvent used in the experiment never exceeded 0.5%.

### Mammalian Cell and Parasites

Vero cells (ATCC® CCL-81™) were cultured in complete RPMI supplemented with 10% FBS at 37°C in a 5% CO_2_ humidified atmosphere. Tissue-derived trypomastigotes of *T. cruzi* (Y strain COLPROT 106) were obtained from the supernatant of infected Vero cells cultured with RPMI (Gibco – Thermo Fisher scientific, Waltham, MA, United States) supplemented with penicillin (100 units ml^−1^) and streptomycin (70 mg ml^−1^; Sigma-Aldrich, St. Louis, MO, United States), plus 10% heat-inactivated fetal bovine serum (FBS) at 37°C in a 5% CO_2_ humidified atmosphere.

### Effects of LQB303 on Intracellular Amastigotes

For infection assays, RAW264.7 macrophages (1.0 × 10^6^ macrophages/well) were seeded and incubated at 37°C and 5% CO_2_ for 24 h. Non-adherent cells were removed; the cultures were washed with phosphate-buffered saline (PBS, 100 mM phosphate buffer and 150 mM NaCl, pH 7.4) and then infected with trypomastigotes, Y strain (MOI 10: 1). After 3 h of interaction, the noninternalized parasites were removed by washing with PBS. The cells were then incubated with or without LQB303 in fresh complete DMEM (hgDMEM; Gibco – Thermo Fisher scientific, Waltham, MA, United States) supplemented with penicillin (100 units ml^−1^) and streptomycin (100 mg ml^−1^) (Sigma-Aldrich, St. Louis, MO, United States) and sodium bicarbonate (3.7 g/L) for 48 h. Benznidazole (50 μM) was used as the standard trypanocidal drug. The cells were stained by quick Romanowsky-type stain (Panotic LB) and examined under light microscopy. The percentage of infection and the number of intracellular amastigotes were quantified using light microscopy (not shown). The infection index was determined by the percentage of infected cells multiplied by the number of amastigotes per cell. The infection index was used to calculate the IC_50._ The IC_50_ values were calculated by fitting the dose response curves with nonlinear regression analysis using the “(inhibitor) vs. normalized response” model of GraphPad Prism 5. All experiments were carried out in triplicate and repeated three times, and the results are presented as the average (±standard error).

As a control, uninfected macrophages (2.5 × 10^5^ cells/well) were also treated with the LQB303 for 48 h, and their toxicity was evaluated by the alamarBlue (Thermo Fisher Scientific Waltham, MA, United States) assay (Roman et al., 2008). The reaction was analyzed at 570 nm ƛ_emission_, using the 600 nm absorbance as the normalization value. The CC_50_ values were calculated by fitting the dose response curves with nonlinear regression analysis using the “(inhibitor) vs. normalized response” model of GraphPad Prism 5. All experiments were carried out in triplicate and repeated three times.

### Amastigogenesis Assays

The differentiation process of trypomastigotes into amastigotes was induced by low pH according to the method described by Paula et al. (2020). Tissue culture-derived trypomastigotes were incubated at 5.0 × 10^6^ cells ml^−1^ in hgDMEM at pH 5.0 and 37°C in a 5% CO_2_ humidified atmosphere. After 24 h, almost 100% of the parasites showed amastigote morphology (rounded morphology).

### LQB303 Activity on Extracellular Amastigotes

To access the trypanocidal effect of LQB303 on extracellular amastigotes, parasites were centrifuged at 3000 × *g* for 5 min, and the pellet was suspended in hgDMEM at pH 5.0 plus FBS 10%. The amastigote suspension was seeded into 96-well microplates at a density of 1.0 × 10^7^ cells/well and treated with increasing concentrations of LQB303 for 24 h. After this time, cell viability was assessed by the MTT method (Mosmann, 1983). The IC_50_ value was calculated by fitting the dose response curves with nonlinear regression analysis using the “(inhibitor) vs. normalized response” model of GraphPad Prism 5. All experiments were carried out in duplicates and repeated three times, and the results are presented as the average (±standard error).

### Flow Cytometry

Extracellular amastigotes were treated with 3 μM LQB303 for 24 h in hgDMEM pH 5.0 medium plus 10% FBS for proliferation. The mitochondrial membrane potential (ΔΨm) was assessed using tetramethylrhodamine methyl ester (TMRM, Invitrogen Corporation Carlsbad, California, United States), a cationic lipophilic fluorescent probe by flow cytometry on a Gallios apparatus with a 488-nm ion-argon laser. Parasites were incubated with 50 nM TMRM for 30 min, followed by the addition of carbonyl cyanide p-(trifluoromethoxy) phenylhydrazone (FCCP, Sigma-Aldrich, St. Louis, MO, United States) 5 μM for 5 min in order to measure the ∆Ψm by flow cytometry. The F_TMRM_/F_FCCP_ ratio was used to normalize the results from the triplicate experiments, where F_TMRM_ is the median fluorescence intensity of TMRM (F_maximal_) and F_FCCP_ is the median fluorescence in the presence of FCCP (F_minimal_; Carranza et al., 2009). All experiments were repeated two times, and the results are presented as the average (±standard error).

### Oxygen Consumption Rates

In the respirometry assays, the extracellular amastigotes were treated with 3 μM LQB303 for 24 h in hgDMEM pH 5.0 media plus 10% FBS for proliferation. Oxygen consumption rates (OCR) of extracellular amastigote (5.0 × 10^7^ parasites/chamber) were evaluated by high-resolution respirometry (Oxygraph-2 K; OROBOROS Instruments, Innsbruck, Austria) with a constant temperature of 37°C throughout the experiment under continuous stirring. Both the oxygen concentration and flux were recorded using DatLab software 5.1.1.9 (Oxygraph-2K-OROBOROS Instruments, Innsbruck, Austria). Subsequently, LEAK respiration was stimulated after the titration of the ATP synthase inhibitor oligomycin (Sigma-Aldrich, St. Louis, MO, United States). The noncoupled state of maximum respiration was induced by the addition of up to 3 μM FCCP, thus allowing the maximal capacity of the electron transfer system to be measured. Respiration was inhibited by the addition of 2 μg/ml Antimycin A, a complex III inhibitor (AA, Sigma-Aldrich, St. Louis, MO, United States) to determine residual oxygen consumption (ROX). The physiological OCR (Routine), the electron transport system maximal capacity (ETS) data were calculated by subtracting the ROX consumption values from the initial OCR and after the addition of FCCP, respectively. The proton leak was calculated after the addition of oligomycin, and the ATP-linked OCR was obtained by subtracting the routine respiration from the proton leak OCR. Finally, the spare respiratory capacity (SRC) was calculated by subtracting ETS from routine respiration (Gnaiger, 2014).

In order to access the non-infected cells respirometry, Vero cells were incubated in the presence or absence of 6 μM LQB303. Forty-eight hours later, 1.5 × 10^6^ cells/chamber were evaluated by high resolution respirometry. Oligomycin (2 μg/ml) was added to achieve LEAK respiration, followed by the titration of up to 3 μM of FCCP to obtain the noncoupled respiration state and the maximum respiration of the electron system. Mitochondrial respiration was inhibited by the addiction of 0.5 μM Rotenone, a complex I inhibitor (Sigma-Aldrich, St. Louis, MO, United States) and 2 μg/ml AA, achieving ROX. Protein concentration was determined by the Lowry method (Lowry et al., 1951) using bovine serum albumin as the standard. All experiments were repeated at least three times, and the results are presented as the average (±standard error).

### Statistical Analyses

All the data presented here were derived from 2–5 independent experiments. Statistical evaluation was performed using GraphPad Prism 5. One-way ANOVA and the Student’s *t* test were used to analyze the statistical differences between the groups. Differences were considered as statistically significant at *p* < 0.05.

## Results

### LQB303 Impairs the Proliferation of *Trypanosoma cruzi*

In order to determine the possible trypanocidal effect of LQB303 on amastigotes of *T. cruzi*, we verified the effect on the proliferation of extracellular and intracellular amastigote forms. The effect was evaluated at different concentrations, after 24 and 48 h of incubation, respectively, at 37°C. As shown in Table 1, LQB303 greatly impaired *T. cruzi* proliferation. The concentration corresponding to 50% of growth inhibition (IC_50_) was calculated, with IC_50_/24 h values of 3.3 ± 0.127 μM for the extracellular amastigotes and IC_50_/48 h 2.6 ± 1.4 μM for intracellular amastigotes. We also calculated the CC_50_ for non-infected macrophages (770.12 μM), showing that this compound was only toxic to mammalian cells in concentrations 296.2 times (SI) greater than the intracellular amastigotes IC_50_. These results indicate that LQB303 has relevant activity against the proliferative phase of the parasite even at low concentrations.

**Table 1:** Trypanocidal activity of LQB303 on different forms of *Trypanosoma cruzi*.

Compound	IC_50_/24 h [μM]	IC_50_/48 h [μM]	CC_50_^c^	SI^d^
	Extracellular amastigote	Intracellular amastigote^a^^,^^b^		
LQB303	3.3 ± 0.127	2.6 ± 1.4	770.12	296.2

a Intracellular amastigotes in macrophages.

b IC_50_/48 h of intracellular amastigotes was based on the infection index (percentage of infected host cells multiplied by the number of parasites per cell).

c CC_50_ (drug concentration which reduced 50% of macrophage viability).

d SI (selectivity index) = CC_50_/48 h/IC_50_/48 h for intracellular amastigotes; average ± standard deviation of at least three independent experiments.

### LQB303 Collapses the Mitochondrial Membrane of *Trypanosoma cruzi*

**Figure 1:**
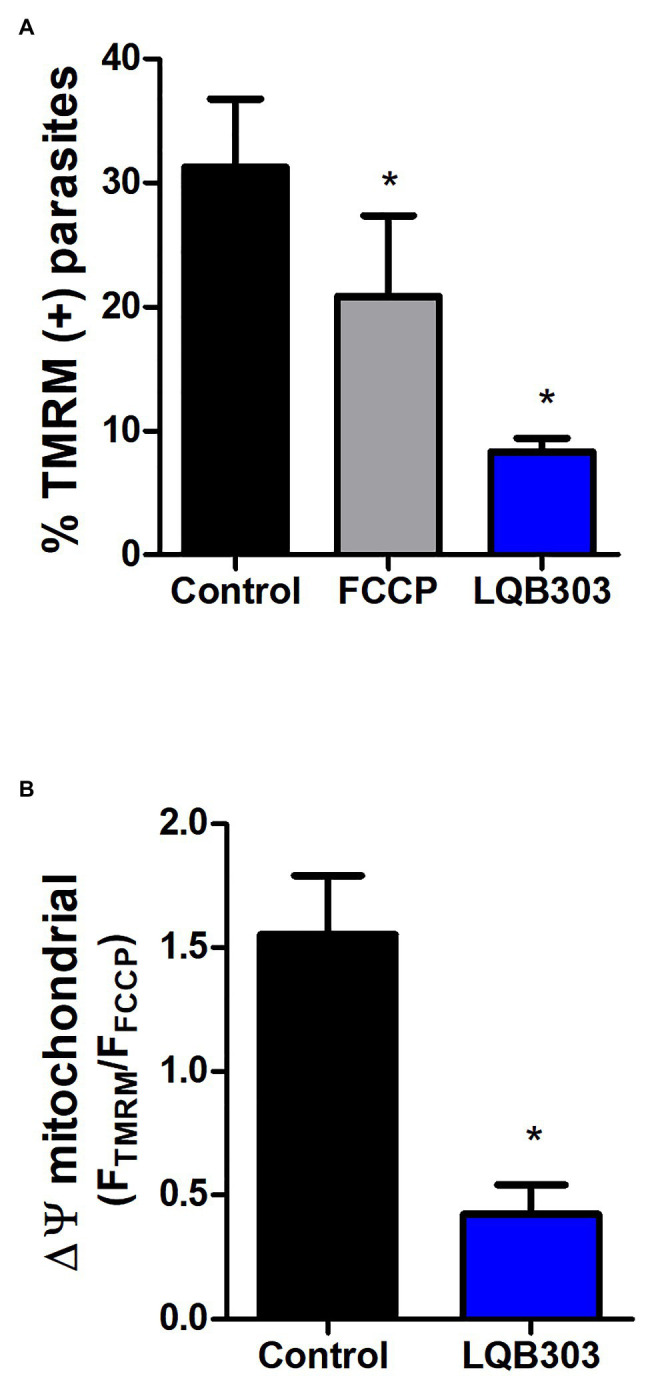
Effect of LQB303 on extracellular amastigotes mitochondrial membrane potential (∆Ψm). Extracellular amastigotes of *T. cruzi* were pre-incubated with LQB303 for 24 h. **(A)** Parasites were incubated with 50 nM TMRM for 30 min, following with the addition of FCCP 5 μM for 5 min to measure ∆Ψm by flow cytometry. Then the percentage of TMRM-labeled parasites was plotted in the presence or absence of 3 μM LQB303 and 5 μM FCCP. **(B)** The F_TMRM_/F_FCCP_ ratio was used to estimate the ΔΨm, where F_TMRM_ is the mean fluorescence intensity of TMRM (F_max_) and F_FCCP_ is the mean fluorescence in the presence of the uncoupler FCCP (F_min_). All experiments were expressed as median ± SD of four experiments. One hundred thousand cells were quantified in each experiment, ^*^p < 0.05 when compared to the control group by one-way ANOVA test (Tukey post-test).

Once we determined the trypanocidal activity of LQB303, we then investigated if this derivative compound conserved the PBN activity in the mitochondria (Wen et al., 2010). Thus, we evaluated the mitochondrial membrane potential (ΔΨm) of amastigotes submitted to or not 3 μM of LQB303 and loaded with the fluorescent probe TMRM, which accumulates in the energized mitochondria. Flow cytometry data demonstrate that LQB303 induced a significant decrease in TMRM positive extracellular amastigotes (73%; Figure 1A). The uncoupler FCCP (5 μM) was used as the mitochondrial depolarization control. The F_TMRM_/F_FCCP_ was plotted as ΔΨm and indicated that LQB303 significantly induced the depolarization of the ΔΨm by about 72% compared to the control parasites, leading to a collapse of the mitochondria inner membrane potential (Figure 1B), confirming our hypothesis that LQB303 targets the mitochondria of the parasite.

### LQB303 Decreases Oxygen Consumption Rates in Extracellular Amastigotes

**Figure 2:**
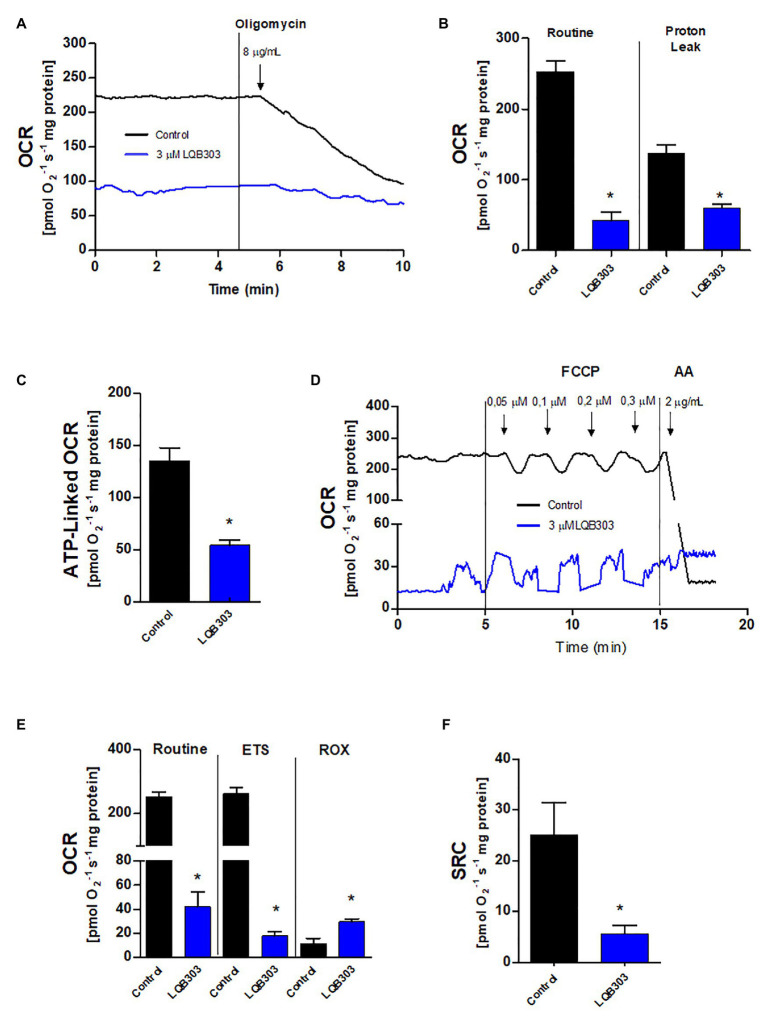
Effect of LQB303 on the oxygen consumption rates of extracellular amastigotes. Extracellular amastigotes (5 × 10^7^ cells/chamber) were incubated with LQB303 IC_50_/48 h at 37°C and then submitted to high-resolution respirometry. **(A)** Representative oxygen consumption rate (OCR) traces of extracellular amastigotes. Where indicated 8 μg/ml oligomycin was added. **(B)** Routine OCR and Proton leak after the addition of 8 μg/ml oligomycin. **(C)** ATP linked OCR was calculated by subtracting basal oxygen consumption rates after adding oligomycin. **(D)** Representative OCR traces of extracellular amastigotes. Where indicated increasing concentrations of up to 0.3 μM FCCP were added. **(E)** Maximal oxygen consumption after ionophore FCCP titration. ROX = residual respiration after the addition of 2 μg/ml AA. **(F)** Spare respiratory capacity (SRC) was estimated as the difference between maximal and routine OCR. All data are presented as average ± SE of at least three independent experiments, performed in duplicate, ^*^p < 0.05 when compared to the control group by Student’s *t* test.

Since LQB303 decreased the mitochondrial membrane potential, resulting in the collapse of the ΔΨm, we continued to investigate the impact of LQB303 on the amastigotes mitochondrial physiology. Therefore, we incubated the parasites with 3 μM of LQB303 for 48 h and then submitted the amastigotes to high resolution respirometry. Figure 2B shows that the pre-incubation of the amastigotes with the compound for 48 h greatly impaired the cells routine oxygen consumption rate (OCR; 42.1 ± 12.3 pmols/s/mg protein) by about 83% compared to control parasites (252.4 ± 15.7 pmols/s/mg protein). Subsequently, we compared the proton leak (after the addition of oligomycin) of treated (59.8 ± 5.7 pmols/s/mg protein) and non-treated parasites (136.9 ± 12.6 pmols/s/mg protein) and observed a decreased of 56% in the treated amastigotes proton leak compared to control parasites (Figures 2A,B). This decrease in the proton leak led to a significant drop (60%) of the ATP-linked OCR of LQB303-treated parasites (54.2 ± 4.8 pmols/s/mg protein) compared to the non-treated amastigotes (135.1 ± 12.8 pmols/s/mg protein; Figure 2C).

Ruas et al. (2016) showed that the maximum oxygen consumption rate can be underestimated when the proton ionophore is added after the use of oligomycin. Therefore, we evaluated the maximum electron transfer capacity (ETS) by titration of the uncoupler FCCP in the absence of the ATP synthase inhibitor oligomycin (Figure 2D). The incubation of extracellular amastigotes with LQB303 for 48 h also led to significantly impair the maximum electron transfer capacity (18.1 ± 3.4 pmols/s/mg protein), compared to the non-treated parasites (261.9 ± 18.1 pmols/s/mg protein), reaching a 93% decrease of the ETS of LQB303-treated amastigotes compared to control (Figures 2D,E). Figure 2F shows that LQB303 also diminished the spare respiratory capacity (SRC) of treated amastigotes by about 77% (5.7 ± 1.6 pmols/s/mg protein) compared to the non-treated parasites (25.1 ± 6.3 pmols/s/mg protein). The residual oxygen consumption (non-related to the electron transport system) was achieved after the addition of AA, a classical inhibitor of Complex III. LQB303 treated amastigotes presented greater ROX (29.6 ± 2.3 pmols/s/mg protein), compared to control (11.6 ± 4.4 pmols/s/mg protein), by 2.6 times (Figures 2D,E). This demonstrated the severe impairment of the mitochondrial physiology of the amastigotes after exposure to LQB303 IC_50_.

### LQB303 Does Not Affect the Mitochondria of Mammalian Cells

**Figure 3:**
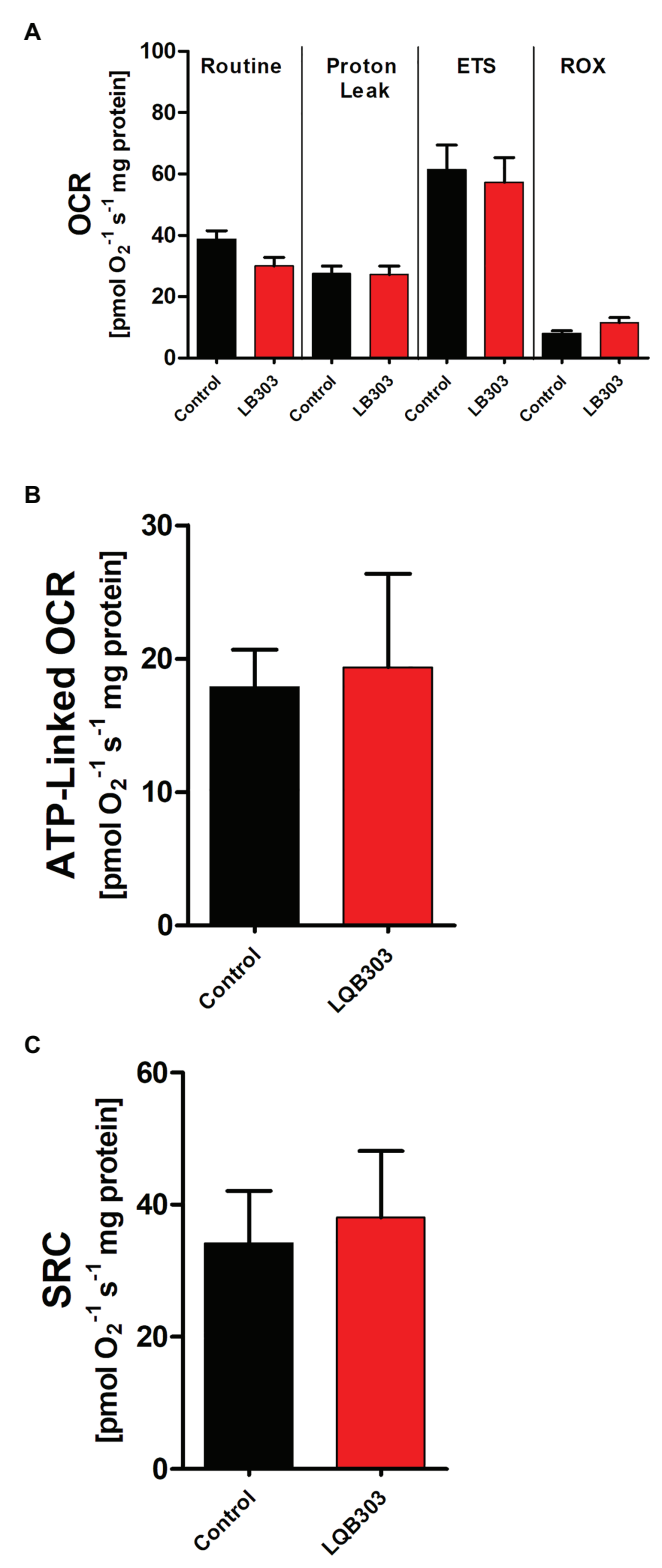
Effect of LQB303 on oxygen consumption rates of non-infected Vero. **(A)** Non-infected VERO cells were cultured in RPMI medium supplemented with 10% FCS and were incubated in the presence or absence of 6 μM LQB303 for 48 h and were assessed by high resolution respirometry. The cells were subjected to 2 μg/ml oligomycin, increasing concentrations of FCCP up to 3 μM, 0.5 μM Rotenone, and 2 μg/ml AA. **(B)** ATP-linked OCR was calculated by subtracting routine oxygen consumption rates after adding oligomycin. **(C)** The spare respiratory capacity was calculated by subtracting the routine from the ETS. All data are presented as average ± SE of five independent experiments, performed in duplicate. There was no statistical difference when compared to the control group by Student’s *t* test.

Since amastigotes proliferate inside nucleated mammalian cells, we investigate if LQB303 could affect the mitochondrial physiology of non-infected mammalian cells using high-resolution respirometry. Therefore, VERO cells were submitted to two times the IC_50_ of extracellular amastigotes (6 μM) for 48 h. Figure 3A shows that non-treated VERO cells, treated with twice the concentration that is enough to greatly impair amastigotes mitochondrial physiology, maintained comparable routine, and ETS and ROX oxygen consumption rates. LQB303-treated Vero cells also showed unaltered ATP-linked OCR and SCR (Figures 3B,C), showing the extreme specificity of LQB303 to *T. cruzi* mitochondrion.

## Discussion

Chagas disease was discovered in 1909, and in spite of that has limited clinical treatment. The two drugs available have serious side effects, variability in treatment and have low efficacy in the chronic phase of the disease, where parasitic persistence increases the risk of heart damage. Therefore, the elimination of the parasite must be done in the early stages of the disease (Wilkinson and Kelly, 2009; Aldasoro et al., 2018). The BENEFIT trial, a prospective and randomized study, showed the limitations of benznidazole in patients with established chronic infection, as there was no significant reduction in the progression of cardiomyopathy and mortality in the advanced cardiac stages (Morillo et al., 2015; Pecoul et al., 2016).

Therefore, there is an urgent need to discover new treatments for Chagas disease. LQB303 reduced the number of both the extracellular and intracellular amastigotes of *T. cruzi in vitro*, and this inhibitory effect on parasite growth was irreversible. Recently, another PBN derivative, LQB123 showed trypanocidal activity against the intracellular amastigotes and other clinically relevant forms of the parasite (Cupello et al., 2017). When comparing the IC_50_/48 h for amastigotes incubated with LQB123 (188 μM) with parasites challenged with LQB303 (3 μM), we observed that LQB303 was 63 times more active than LQB123. Moreover, this result also shows that the new modification in the PBN molecule maintained the low toxicity to mammalian cells with a high selectivity index (>296.2; Table 1).

Unlike mammalian cells, the members of the order Trypanosomatida exhibit a single mitochondrion with peculiar functional and morphological characteristics, such as the presence of a dysfunctional complex I insensitive to rotenone, and the presence of a specific arrangement of mitochondrial DNA, named as kinetoplast. Due to its uniqueness, this organelle has become an attractive target in parasites for new drugs (Fidalgo and Gille, 2011; Menna-Barreto and de Castro, 2014).

Natural sources have been widely used to lead the discovery of new compounds for neglected diseases (Ioset and Chang, 2011; Ndjonka et al., 2013). Indeed, there are many natural bioactive molecules used to target the mitochondrial metabolism of these parasites. A substantial number of trypanocidal drugs, including compounds clinically used and others under investigation, have the mitochondrion as at least one of their targets. The mitochondrion as a target has been extensively explored in *Leishmania*, strongly correlating the organelle to the survival of the parasites. Apigenin is a good example of a well-studied naturally occurring molecule found in many plants with documented antiprotozoal activity (Singh et al., 2014). This bioactive flavone promoted mitochondrial dysfunction, ROS production, and parasite death in *Leishmania amazonensis in vitro* (Fonseca-Silva et al., 2015) and *in vivo*, and it efficiently treated cutaneous leishmaniasis (Fonseca-Silva et al., 2016). Amphotericin B causes permeability of membranes with a rapid decrease of the mitochondrial membrane potential followed by a simultaneous induction of plasma membrane permeability (Lee et al., 2002). Naphthoimidazoles are another class of molecules that target the mitochondrion. β-lapachone derivatives directly impair *T. cruzi* electron transport system, resulting in increased ROS production and parasite death (Bombaça et al., 2019).

In fact, the analysis of the mitochondrial function of LQB303-treated extracellular amastigotes showed a loss of mitochondrial membrane potential (Figure 1). In addition, among the apoptotic phenotypes, mitochondrial membrane potential (ΔΨm) loss and blebs in the plasma membrane are the most relevant (Menna-Barreto, 2019). We additionally studied the activity of LQB303 on the mitochondrial physiology of amastigotes and observed that the parasites treated with IC_50_/24 h suffered an important decrease in oxygen consumption rates compared to the control parasites.

The mitochondrial flow of electrons through the electron transport system was reduced after the addition of oligomycin in non-treated amastigotes. FCCP interrupts ATP synthesis by transporting protons across the cell membrane, increasing the rate of oxygen consumption to the maximum (Lerchner et al., 2019). However, these effects were not observed in the LQB303-treated amastigotes, since the maximum electron transfer capacity did not exceed basal levels (Figures 2B,E). LQB303 induced mitochondrial dysfunction, which greatly impaired the ATP-linked OCR and the spare respiratory capacity of amastigotes (Figures 2C,F). This result, allied with increased ROX (Figure 2E) in treated parasites correlates with the loss of the mitochondrial membrane potential, strongly suggesting mitochondrial dysfunction, which could culminate with the decreased proliferation.

Another example of mitochondrial dysfunction resulting in parasite death is LQB-118-treated Leishmania. LQB-118 is a widely studied pterocarpanquinone that has demonstrated potent antileishmanial activity against *Leishmania amazonensis* (da Cunha-Júnior et al., 2011) as well as *L. braziliensis* inducing oxidative stress, mitochondrial dysfunction, ATP depletion, and DNA fragmentation, controlling the lesions of infected hamsters (Costa et al., 2014). The fact that LQB303 showed no interference in VERO cells viability or impaired the mitochondrial bioenergetics indicates the specificity of LQB303 for amastigotes mitochondria and is an encouragement to continue studies with this promising molecule.

In summary, our results suggest promising trypanocidal activity of LQB303, since it leads to specific inefficiency of amastigotes bioenergetics, but does not influence the host cell energy metabolism or growth, making this compound an attractive alternative for Chagas disease treatment. Further biochemical studies are needed to fully elucidate the mode of action of LQB303.

## Data Availability Statement

The raw data supporting the conclusions of this article will be made available by the authors, without undue reservation.

## Author Contributions

NN and CM: conceptualization. NN, MP, and FS: data curation. NN, MP, CM, FS, and JP: formal analysis. NN and MP: funding acquisition, project administration, and validation. NN, MP, FS, and AD: investigation. CM, FS, JP, SN, DC, PC, and AD: methodology. NN, MP, and AD: supervision.

### Conflict of Interest

The authors declare that the research was conducted in the absence of any commercial or financial relationships that could be construed as a potential conflict of interest.
